# Epidemiology of Muay Thai fight-related injuries

**DOI:** 10.1186/s40621-016-0095-2

**Published:** 2016-12-12

**Authors:** Stephen Strotmeyer, Jeffrey H. Coben, Anthony Fabio, Thomas Songer, Maria Brooks

**Affiliations:** 1 University Center for Social and Urban Research (UCSUR), University of Pittsburgh, 3343 Forbes Ave, Pittsburgh, PA 15232 USA; 2 Injury Control Research Center, West Virginia University, PO Box 9151, Morgantown, WV 26506-9151 USA; 3 Department of Epidemiology, University of Pittsburgh, 130 DeSoto Street, Pittsburgh, PA 15261 USA

**Keywords:** Fight, Injury, Muay Thai, Combat sport

## Abstract

**Background:**

Muay Thai is a combat sport of Thailand that uses stand-up striking along with various clinching techniques. Currently, little is known about the injuries and risk factors for injuries among Muay Thai fighters. Gaining more insight into the nature and frequency of injury in this sport provides part of the overall sports injury picture, within the larger burden of injury as a public health issue. Generating this information is a critical first step toward the broader goal of improving the health and safety of Muay Thai fighters engaged in competition.

**Methods:**

This study is based upon a survey of 195 Muay Thai fighters. Participants were asked to complete a retrospective web survey on fight-related injuries. Regression analyses were conducted to determine whether injuries during sanctioned fights were related to factors such as fight experience, use of protective equipment, and injury history.

**Results:**

Participants were aged 18 to 47 years old (median 26), predominantly male (85.9%), and white (72.3%). Respondents were professional (*n* = 96, 49.2%) and amateur (*n* = 99, 50.8%). Fighters reported a mean fight experience of 15.8 fights. Of the 195 respondents, 108 (55.4%) reported sustaining an injury during the most recent fight. The primary body region injured was the extremities (58%) versus the head, with a lower amount of self-reported concussions (5.4%). Nearly 2/3 (66.7%) of all injured fighters reported that the injury did not interfere with the bout outcome. Nearly 25% reported they missed no training time as a result of the injury. Injuries were related to professional fighter status (OR = 2.5, 95% CI = 1.4–4.5), fight experience (OR = 2.7, 95% CI = 1.5–4.9), weight class (OR = 0.923 heavy versus light, 95% CI = .86–.99), age (OR = 0.90 > 26 versus ≤26, 95% CI = .85–.95), use of protective equipment (OR = .46, 95% CI = .26–.82) and previous injury (OR = 1.81, 95% CI = .98–3.3). Lighter, younger, and more experienced fighters were at increased odds for injury within this sample.

**Conclusions:**

We identified a preliminary fight-related injury rate and identified fighter characteristics (experience level, protection level, and previous injury) associated with increased odds for fight-related injury outcome. While rigorous research into causality is required, these data provide plausible information that may be used to reduce injury outcomes among fighters.

## Background

Combat sports, such as Mixed Martial Arts (MMA), Ultimate Fighting Championship (UFC) and Muay Thai, have increased in popularity over the last decade. Cage fighting has evolved from a small-time fringe spectacle banned in many states to the fastest-growing spectator sport in America (Gottschall 2016). While combat sports involve two combatants fighting under distinct rules of engagement, due to the nature of striking, throwing or immobilizing an adversary, combat sports are generally considered more dangerous and injury prone compared to other athletic activities (Binner 2014; Lystad et al. 2014).

One sport gaining recent global recognition is Muay Thai, a combat sport from Thailand that uses stand-up striking along with various clinching techniques to throw an opponent to the ground. Muay Thai is referred to as the “Art of Eight Limbs” because it makes use of punches, kicks, and elbow and knee strikes, thus using eight “points of contact,” as opposed to “two points” (fists) in boxing and “four points” (hands and feet) used in other more regulated combat sports, such as kickboxing. Muay Thai also allows strikes to all three body regions (head, body, legs). Despite its increasingly popularity and being a preferred style for stand up training among MMA fighters, Muay Thai yields scant epidemiologic study on fighter injuries. With this surge in popularity, many other combat sports that comprise the mix in styles of MMA are experiencing increased levels of participation over the past several years (Lund et al. 1994). It is imperative to establish surveillance systems that adequately collect injury information to quantify the frequency and nature of recorded injuries and to analyze the determinants and causal factors associated with injury. The primary aim of this study was to describe the frequency and severity of Muay Thai fight-related injuries among a sample of professional and amateur fighters. A secondary aim was to explore the underlying demographic factors associated with the reported injury outcomes.

## Methods

Surveillance of Muay Thai fight injury was conducted from April 6, 2010–January 17, 2011 using a logic driven, web-based survey. The survey was a 40-item questionnaire constructed with face validity by established Muay Thai experts, including coaches (4), officials (2), fighters (5) and ringside physicians (6) and pilot-tested on a subset of fight participants (*n* = 27) prior to deployment. Participants within the sport added subjective measure of the extent to which the survey content appeared reasonable. The survey collected basic details on the elements of the fight itself and, depending on whether or not an injury was reported, additional injury-related questions were asked (e.g., nature, mechanism, body region, severity). Complete surveys were collected from 195 respondents from the United Kingdom and North America that participated in sanctioned Muay Thai fights. Fighters were sampled from high profile fight regions and approached at prominent events. In these regions, fight events are governed by the athletic commission and sanctioned under established rules for legal competition. Fighters were recruited for participation in person or via email from the research team or the sanctioning body. We employed one of the most common types of nonprobability sampling, a convenience sample. Fighters were recruited using emails and social media to announce the survey by several sanctioning bodies in the United States, Canada and the UK. Anyone with access to the URL would be able to participate. We used individuals available within the above mentioned high profile regions rather than attempting selection from an unknown population. This resulted in 154 respondents that completed the survey. A second group was recruited using a targeted approach. Nine fight events were randomly drawn over the study period, and ten fighters were then randomly drawn for recruitment. Of the 90 approached fighters, 41 completed the survey (45.5%).

Fighters were asked to complete the survey within a few days of the fight in our targeted sample and for the convenience sample, whether a painful injury occurred in a fight within the past 6-weeks. If no, then they were asked whether they had sustained a fight-related injury within the previous 6-months. Fighters were instructed to consider only fight-specific injuries (in the ring/location of injury), rather than those sustained during training prior to the fight. Additionally, if multiple injuries were incurred in a bout, fighters were asked to classify the primary injury: “If you sustained multiple injuries during the fight, we ask you to think of the most severe and single injury.” If fighters reported more than one fight in the 6-month window with multiple fights resulting in injury, only the most recent fight within the recall period was the focus of the injury-related questions.

The surveillance included variables consistent with the International Collaborative Effort (ICE) on Injury Statistics minimum basic dataset for injury monitoring in addition to elements associated with the injury incident (Hosmer Jr et al. 2013). Cases of fight-related injury for each fighter over the study period (2010) were identified from the survey data. Additional data collected on all participants included the number of total fights fought and what the fighter’s rank or status is, namely amateur or professional (Table 1). For operationalizing, fighting experience was assessed from two perspectives: we examined the binary (professional versus amateur) and the continuous (number of total fights) variables. Anecdotally, many fighters may never opt to fight professionally, therefore accumulating considerable experience, whereas others may jump to the pro ranks prematurely with relatively little time in the sport. The survey also gathered information regarding types of protective equipment worn during the fights. We classified the levels of protection as 1) gloves only or 2) gloves, shin pads, and headgear. Finally, the survey assessed pre-fight injury history by examining whether or not the fighter entered the fight with or without an injury derived from a previous bout: 1) how many fights in the prior 6 months had participants fought and sustained another injury? Or 2) was the incidence of the fight-related injury in question entirely new or a recurrence or aggravation of a previous injury?

**Table 1:** Key variables assessed in the survey of Muay Thai fight injury frequency

Date of injury incident	Time of injury incident	Location of injury incident	Fight decision
Fighter class Professional/Amateur	Number of total fights experience	Fight classification	Fight description (open-ended)
Injury Severity	Lost training time	Nature of injury	Body part injured
Mechanism of injury	Injury incident report (open-ended)	Initial treatment	Protective equipment worn
Age	Gender	Race/Ethnicity	Fighter weight class

Frequency distributions were used to summarize and present the data collected on the variables of interest, including time, place, experience level, severity, nature and mechanism, age, gender, race, fight outcome, weight class and equipment worn. Table 2 outlines the characteristics of the survey respondents and distribution of these key variables. Bivariate analyses, using chi-square statistics and t-tests, were performed to assess the relationships between predictor variables as well as their relationships to the outcome variable (injury).

**Table 2:** Demographic characteristics of Muay Thai fight respondents (*n* = 195)

	*N*	%
Gender		
Male	165	85.9
Female	27	13.8
Age (n = 192)		
18–24	65	33.9
25–34	107	55.7
≥ 35	20	10.4
Weight category (*n* = 194)		
Light (≤147 lbs.)	67	34.5
Heavy (>147 lbs.)	127	65.1
Fight Experience (median = 11)		
1–9 fights	90	46.2
10–15	29	14.9
≥ 16 fights	76	38.9
Fighter Level		
Amateur	99	50.8
Professional	96	49.2
Fighter Protection		
Gloves only	110	56.4
Gloves plus (additional headgear, shin pads)	85	43.6

Unadjusted logistic regression models were created to assess whether fight-related injury (yes versus no) as the outcome variable was associated with fight experience, both using a continuous variable (# total fights fought) and a dichotomous variable (amateur versus professional fighter). We also assessed whether fight-related injury was related to wearing protective equipment, comparing two levels of protection (gloves versus gloves, headgear, and shin pads). Finally, we examined whether or not having sustained an injury before the fight impacted the incidence of injury during the surveyed fight.

Multivariable logistic regression was used to evaluate the association between reporting a pre-existing injury when entering the bout and incurring a subsequent injury during the fight as well as other predictor variables. A *p* value < .10 was used to identify variables for the multiple regression model (Gartland et al. 2001). Age was forced into the model as well, due to the fact that aging and the maturation process are internal, unmodifiable risk factors for sports injury, as physical attributes such as strength, speed and flexibility diminish. Backwards stepwise regression procedure was used to remove variables based on the exit criterion (*p* > 0.10) (Gartland et al. 2001). Statistical analysis was performed using SPSS v21.

## Results

### Descriptive epidemiology of Muay Thai fight injury

Key descriptive characteristics of the 195 fight respondents are shown in Table 2. The sample was primarily comprised of young male fighters under age 35. About one-half were professional fighters and the median number of prior Muay Thai fights in the sample was eleven. Nearly one-half (43%) wore protective equipment (head gear and/or shin pads) during the sampled fight.

Among the 195 respondents, 108 (55.4%) reported sustaining an injury during the fight, while the remaining 87 (44.6%) reported no incidence of injury. The overall injury rate was 55 injuries per 100 fight exposures. The fighters reporting injury were predominantly professional (59%, *n* = 64), did not wear protective padding other than gloves (65%, *n* = 70), and did not enter the fight with a reported previous injury (59%, *n* = 64). When asked about the nature of the primary injury from the fight, respondents reported that the majority of these were bruises or contusions (38.7%) followed by cuts or lacerations (14.4%). The primary body region that was injured were the extremities in more than half of the reported fight injuries (58.6%). There were, comparatively, fewer head injuries (30.6%) (Table 3).

**Table 3:** Characteristics of reported injury in Muay Thai fights (*n* = 108) by nature, mechanism, body region, severity

Nature	Number	Percent
Bruise/Contusion	43	38.7
Cut/Laceration	16	14.4
Swelling/Inflammation	15	13.5
Fracture	14	12.6
Concussion	6	5.4
Sprain, Strain, Overexertion	12	10.8
Mechanism		
Struck by opponent	75	67.6
Collision with opponent	24	12.3
Lifting/Pulling	2	1.8
Other (Struck opponent)	10	9.0
Primary body region injured		
Extremities	65	58.6
Head/Neck	34	30.6
Trunk/Torso	12	10.8
Injury severity		
Injury did not interfere with completion of fight	72	66.7
Injury affected performance, but not completion of fight	7	3.6
Injury did interfere with completion of fight, but not subsequent training/fighting	7	3.6
Injury did interfere with completion of fight and affected subsequent training/fighting	22	11.3

The primary cause or mechanism of the fight injuries was due to being “struck by” the opponent in more than two thirds of the reports (67.6%). Colliding with the opponent caused the next highest proportion of injuries (12.3%). About 10% of reported injuries (indicated as “other, specify”) were a consequence of striking the opponent, versus being struck by the opponent.

The bulk of the injuries reported by the fighters were low in severity. Of the 108 fighters who reported an injury, self-reported severity levels reported ranged from Level 0, where the “injury did not interfere with completion of the fight and had no bearing on outcome” (66.7%) to Level 4, the “injury did interfere with fight and affected subsequent training or fighting” (11.3%) (Table 3). If injured, participants were asked to identify the type of treatment they received to care for the injury. Nineteen fighters reported that no treatment was required. Thirty-five fighters reported using only self-treatment. The remaining 54 fighters sought a range of medical treatments, with most using the Rest, Ice, Compression and Elevation (RICE) protocol (57.4%). Following up with the initial injury treatment, respondents were asked who actually performed the treatment received, if not self-treated. In these instances, the fight trainer (37.5%) initially treated the injury, followed by emergency medical services (23.4%), emergency departments (7.8%), outpatient care (14.1), inpatient care (7.8%), and physical therapy (9.4%).

Six concussions (5.4%) were reported as the primary nature of injury within this sample of Muay Thai fighters. Five of the six concussion events occurred among professional fighters. Those reporting a concussion as the primary injury all indicated the injury interfered with the completion of the fight (all resulting in a stoppage). Thirty-one fighters reported about stoppage, most often the result of cuts (*n* = 7), bruises (*n* = 7), concussion (*n* = 6) or fractures (*n* = 5). Half of the fighters sought medical treatment even after being seen by an emergency medical technician on site. Four out of the six reported that the concussion affected subsequent training and fighting due to taking time off to recover (range: 2 days – 4 weeks).

Of the injured fighters, when asked “How much training time did you miss due to this injury?”, over one third reported they missed no training time as a result of the injury incurred during the fight (33.6%). They did not perceive the injury to impact either completing the fight or the outcome (win, loss, draw). When analyzing the injury severity by the fight outcome, a significant difference was noted, in that the fighters that lost were more likely to report a higher injury severity (*p* = .039).

In addition to lost training time, fighters were queried as to whether or not they had to cancel or postpone a scheduled fight as a result of the injury. Thirty-six (33.3%) of the fighters declared they did not need to cancel a fight, as one was not scheduled. Of the remaining group, 60 of the injured fighters did not need to postpone or cancel (55.6%). Only 12 (11.1%) answered that the fight injury forced them to cancel an upcoming bout.

### Risk factors related to reported injury

The relationship between several fight-related covariates and injury was also assessed in this sample. Both age (OR = 0.90 for >26 versus ≤26; 95% CI = .85–.95) and weight (OR = 0.92 heavy versus light; 95% CI = .86–1.0) were found to be significant factors related to reported fight injury. Reported frequency of injury did not differ by gender.

#### Fight experience

In reports from other combat sports, fight experience has been related to injury outcomes (Binner A. The rise of mixed martial arts. Banned in most of the US states to global athletic phenomenon boasting sell-out events & the sport has taken giant leaps. In: Ajazeera. Sport 2014; Gartland et al. 2005; Zetaruk et al. 2005; Fulton et al. 2014). Thus, a key question in this study was to answer if Muay Thai fight experience was related to reported fight injury, hypothesizing that less experienced fighters would have higher injury frequency and severity. However, in this sample, higher levels of identified fighter experience were associated with a higher reported frequency of injury (OR = 2.7; for >15 fights; 95% CI = 1.5–4.9). This relationship remained significant in a multivariate model (OR = 3.6, *p* < 0.001) with age, weight, gender, fighter protection use, fighter status (amateur or pro), and previous reported injury. Neither protection level nor previous injury was found to be significant in a stepwise model and both were removed from the final model.

Fight experience was also examined as a continuous variable (number of fights) and remained significantly associated with reported injury in a multivariate assessment. Each additional fight was associated with a 1.05 greater odds of sustaining a fight-related injury (*p* = .001), adjusting for age and gender (Table 4).

**Table 4:** Regression Analysis of the relationship between injury and fight experience in 190 Muay Thai fighters

	Beta Value	Standard Error	Odds Ratio	95% Confidence Interval
Fight experience (per fight)	.04	.01	1.05	1.02–1.07
Age (>26)	−1.26	.33	.29	.15–.54
Gender (Female vs. Male)	1.04	.48	2.83	1.10–7.30
Constant	−.97	.61	.38	

Variables entered on STEP 1: Fight experience (CV), Pro v. Amateur, Protection Level, Preexisting Injury, Weight, Age (>26), Gender (Female v. Male)

#### Protective equipment

A critical question in Muay Thai involves the role of protective equipment in the prevention of injury. Several fighters use head gear, gloves, and/or shin pads for the perceived purpose of reducing injury in fights. The relationship between fight-related injury and the degree of protective equipment worn was examined in this sample. Univariately, a strong association of protective equipment was identified for a reduced frequency of injury (OR = .46; 95% CI = 0.26–0.83). This relationship, however, did not remain when assessed in a backwards stepwise regression model. When adjusting for age and weight and including fight experience and preexisting injury in the model, the use of protective equipment was not found to be significant.

#### Previous injury

As much of the injury literature has indicated that history of injury is a strong factor related to subsequent injury (Bledsoe 2009), we sought to determine if fight-related injury was related to pre-existing injury. It was hypothesized that injured fighters compared with non-injured fighters differ with respect to previous fight injury. In this sample, indication of a previous injury prior to the fight was not associated with a reported injury during the fight (OR = 1.8; 95% CI = 0.98–3.3). When adjusting for age, weight and sex, and including fight experience, fighter status and protection level in a multivariate model, previous injury was not related with reported fight injury (OR = 1.86; 95% CI = 0.95–3.67).

## Discussion

In this study, more than half (55.4%) of the Muay Thai fighters reported an injury in their most recent contest. Most of the reported injuries were soft-tissue injuries and lower on the injury severity scale. About 20% of the injuries involved a fracture or concussion. In most circumstances, the injuries were reported as not interfering with the completion of the fight, nor its outcome. Overall, the fighters reporting a higher frequency of injury were younger, female, had more ring experience, and were professional caliber fighters. Previous injury history was not associated with reported fight injuries in this sample.

The lower extremities (55/108, 51%) were the most commonly injured body region injured during fights, as detailed in the injury incident description. In contrast, concussion represented a small proportion of reported injuries. This finding may be due, in part, to the tactics involved in Muay Thai fighting where one can attack multiple targets, including the body and legs, with eight weapons (two hands, two elbows, two knees, and two legs). Given that scoring in Muay Thai awards strong kicks and knees, the whole body may be a primary target compared to boxing, which targets the head most frequently.

These findings are similar, in part, to the results of three previous studies published on Muay Thai (Gartland et al. 2005; Shirani et al. 2010; Gabbe et al. 2003) but differ with regards to the injury definition, severity level, exposure, and competition level (Table 5). Additionally, we were able to compare injured versus uninjured fighters for the first time.

**Table 5:** Summary of Muay Thai injury studies

Author	Injury Definition	Severity Level	Exposure	Experience (Pro vs. Amateur)
Gartland et al. (2001)	Self-report; face-to-face interview	Number of missed training days (enforced absence due to injury)	Contact level: None, Touch Sparring Full contact, Competition	Pro & Amateur
Gartland et al. (2005)	Self-report to medic or referee report	Not specified	Fight	Amateur
Shirani et al. (2010)	Physician referral to maxillofacial surgeon	Required physician treatment; injury screened radiographically	Training & Fight	Pro & Amateur
Strotmeyer (2014)	Self-report Survey	4 Severity levels, time loss	Fight	Pro & Amateur

Compared to previous research regarding Muay Thai injuries, the current study echoed common injury outcomes to the lower extremities from soft tissue contusions (Gartland et al. 2005), and to the head (Shirani et al. 2010). Injuries to the head were the second leading body region injured in the current study and one previous study (Gartland et al. 2005), but the primary outcome in two other studies (Gartland et al. 2005; Gabbe et al. 2003).

This finding lead to speculation that it might be an acceptable norm to incur minor bumps and bruises to the lower extremities which are not perceived to be injuries by the participants (Shirani et al. 2010). Wearing shin pads may conceal minor injuries rendering them undetectable by a referee or medic, leading to under-reporting, whereas noticeable contact to the head, as witnessed by the referee, may have led to increased reporting to the medics for precautionary measures. The current study’s finding of injury to the lower extremities among 55% amateur and 51% professional were comparable to the 64% amateur and 53% professional as well (Gartland et al. 2005). Further, the most common nature of injury in both studies was soft tissue injuries, predominantly contusions.

We did find found slightly more lacerations (20%), followed by fractures (13%) compared to previous research (Gartland et al. 2005) that reported fractures as the second leading nature of injury among professionals. The lacerations in the current study were generally (75%) the result of cuts from elbows to the head. Elbows are a dangerous technique rarely used in training exercises unless wearing heavy padding to reduce the potential danger of being cut. Because previous research (Gartland et al. 2005) included training exercises as an exposure and the absence of this technique in practice could explain the lower incidence of lacerations. Other research among a subset of Muay Thai fighters ranked lacerations were the most common outcome (93.3%) and more injuries were reported among the professionals (86%) compared to the amateurs (42%) (Gabbe et al. 2003). While the current study is not directly comparable, there were more professionals injured (65%) than amateurs (44%) and more head injuries among the professionals (33% vs. 25%). The professionals with head injuries in the current study did report lacerations (57%), injuries to the jaw (14%), concussions with pain (24%), and several eye injuries (5%). Those cut in all cases sought medical treatment, largely for sutures for the lacerations. The current work found that the majority of the facial lacerations to the professional fighters (84%) were from being elbowed, kneed (8%), or punched (8%) by the opponent. It is difficult to compare directly to previous research as it was not specified how, where or when these occurred, only commenting that it resulted from Muay Thai “participation.” (Gabbe et al. 2003) Further, the mechanism of injury was not presented, only the nature of the injury itself within a clinical setting.

We reported similar age ranges (18–47 years) with one study (14–51 years) (Gartland et al. 2005), having identical medians (26 years), but our sample was slightly older than those reported in two other studies (mean 17 and 20 years old) (Shirani et al. 2010; Gabbe et al. 2003).

Females comprised 17% in the current study, similar to 13 (Gartland et al. 2005; Shirani et al. 2010) and 20% (Gabbe et al. 2003). Differences were reported injuries among novice, amateur, and professionals but noted confusion about these definitions, possibly, since training exercises were included (Gartland et al. 2005). Not all participants were fighters, therefore some had difficulty self-identifying their rank or caliber. We looked exclusively at fight exposures, therefore professional or amateur were easier to categorize. Training reflects considerably less intense contact levels deliberately in effort to prevent injury (Table 5). This exposure level difference may account for relatively small percentages of time off from training (7%), defined as 7 days or more compared to 25.9% found in the current study (Gartland et al. 2005).

Information on the mechanism of injury, protective equipment worn and a brief narrative was collected in the present study. Of the 44 injured amateur fighters, 25 incurred injuries to the lower extremities (7 not wearing shin pads; 18 padding worn). The brief narrative description revealed that the majority of these (16 out of 25) were a consequence of being struck by the opponent, who presumably would also be wearing shin pads, as fighters wear the same level of protection in sanctioned fights. Damage inflicted to the lower extremity was reported by fighters while wearing protection, against a similarly padded opponent. This increased level of detail was absent from previous research (Shirani et al. 2010).

That study (Shirani et al. 2010) concluded that younger, less experienced, and heavier fighters were at increased risk for injury. This result is quite different than reported in the current study, which was that younger, more experienced and lighter fighters were at increased risk. One possible reason might be that the previous work (Shirani et al. 2010) did not include professional fighters, and among those amateurs, a lower reported a mean of 3.4 fights was quite different compared to the 16 fights mean, with nearly 50% being professional fighters in this study. A bias was also noted in the previous study in the heavier weight classes due to extremely small sample size (*n* = 4) with a considerably high number of injuries reported (Shirani et al. 2010).

Another possible explanation for why less experienced fighters were at increased risk in the previous study compared to the current work might be due to the level of intensity (Shirani et al. 2010). Younger, experienced professionals are more adept and often driven by fight incentives such as purse or prize money and titles. Professional fighters are considerably more skillful. Coupled with a winning drive, this may lead to more furious efforts when compared to the relative neophytes in the previous study’s sample, who are still learning and honing techniques, both offensively and defensively (Shirani et al. 2010).

One previous study reported an injury rate based on competition minutes recorded at the events, and identified an average rate of 9.1 injuries/100 min of competition (Shirani et al. 2010). In the current study, considering the 44 injured amateurs and bout time fought per fight (3 rounds × 2 min) results in roughly 264 min of competition time. This number would be an overestimate since fights stopped during the round were rounded up, not every bout went the distance, and, although rare, some less experienced amateurs may fight 1.5 min rounds. These numbers result in 16.6 injuries/100 min competition time, slightly higher than and perhaps how the injuries were reported (referee, medic, some self vs. self-report) lead to more over reporting in this current work’s sample.

There were some limitations with our study. Self-selection survey bias exists as fighters who were injured may be more likely to complete a survey targeting injury outcomes, so while only collecting the primary injury and not multiple injuries, we may be overestimating the incidence, particularly compared to other combat sports such as MMA (Bledsoe 2009) (28.6/100) or boxing (Zetaruk et al. 2005) (25/100). Additionally, we employed a non-probability sample, or convenience sample, therefore introducing sample bias and therefore the results are not representative. However, as the population of Muay Thai fighters is less quantifiable, extrapolating back to that target population was not a primary objective but rather to investigate the relationships between several key variables among those sampled.

We defined an acute injury as “painful” physical harm sustained during an actual fight and asked respondents to consider fight-specific injuries (in the ring), rather than those sustained during training prior to the fight. If multiple injuries were sustained, the primary injury of interest was the injury the fighter felt was the most severe. This study did not capture all of the injuries during the fight but focused on a single injury that was self-reported to be the most severe. Additionally, as the case definition concentrated on the pain aspect of the injury, it does not factor in that the mechanism of injury could have started prior to the fight without the emergence of pain. As pain threshold is an entirely subjective phenomenon, some individuals may tolerate higher levels of pain compared to others, therefore resulting in differences in reporting injury according to the current study’s case definition. For example, the identical injury occurring for two different fighters may result in only one reporting the injury based on the definition focusing on reported pain. Additionally, fighters may have not experienced pain with concussion, therefore may not have disclosed an injury which conceivably could lead to underreporting of concussions within this study.

The retrospective nature of the study design introduces the possibility of recall bias. By design, the current study restricted the recall period to a maximum of 6 months, since Gabbe’s publication (Zazryn et al. 2006) found that injury rates over a 1-year time period had perfect recall whether an injury had occurred, with decreasing percentages of participants recalling the exact number, body region or diagnosis. Therefore, it stands to reason that the current study’s injury rate is likely to be accurate, perhaps with decreasing accuracy in the reported total number of injuries, location, and diagnosis obtained from self-reporting. There were two different recall periods, as within the targeted sample, fighters were approached within a week of fighting, compared to the convenience sample that allowed for injury recall up to 6 months. Training injuries were not included in this survey, another limitation, though previous fights with an injury sustained were documented by asking fighters, “How many fights have you had in the past 6 months where you sustained at least 1 injury?”

Despite these limitations, the current study also has several advantages. Our injury criteria were concrete and encompassed a wide range of injuries seen within the combat sports, particularly within Muay Thai. Although not validated, the survey piloted a means of electronic delivery for web-based surveying, which could easily be replicated on a grander scale among more participants. This study was structured as a pilot which could provide areas of focus for further studies. A larger, prospective study with a validated survey and examination of injury rates and patterns with elements related to experience, protection, preexisting injury, length of time in the sport (stratifying for amateur and professional fight exposures), and training activities may then be explored in more detail to help design effective prevention strategies to reduce injury rates and aid Muay Thai grow into a safe and effective sport and recreational activity.

## Conclusions

In summary, we identified a fight-related injury rate of 55 injuries/100 fight exposures, coupled with the characteristics of fighters associated with fight-related injuries; the most common location of injury; the nature, mechanism, and severity of injury; fight level factors such as experience level, protection level, and existence of previous injury associated with injury outcome. While unique associations, they require more rigorous research exploring causal factors. However, the current information can be used by fighters, trainers, and officials who participate directly in the sport to prevent and treat injury. It cannot be overemphasized how important it is for all individuals involved in the decision-making process to be fully informed as to what factors may impact fighter injuries.

## Abbreviations

ICE: International Collaborative Effort
MMA: Mixed martial arts
RICE: Rest, ice, compression and elevation
UFC: Ultimate Fighting Championship
